# Caries-Exclusive and Health-Associated Taxa in the Peruvian Pediatric Salivary Microbiome Identified by Full-Length Nanopore 16S Sequencing: Implications for Ecological Modulation of Dental Caries

**DOI:** 10.3390/microorganisms14091897

**Published:** 2026-08-26

**Authors:** Laly Abigail Rojas Rodríguez, Carlos Michell Gálvez Ramírez, Marco Aurelio Salvatierra Celis, Justo Nilo Balcazar Conde, Margarita Fé Requena Mendizábal, Rocio Del Pilar Bocanegra Arista, Roger Dámaso Calla Poma, Tania Valentina Rosales Cifuentes

**Affiliations:** 1 Escuela Profesional de Odontología, Facultad de Odontología, Universidad Nacional Mayor de San Marcos (UNMSM), Av. Germán Amézaga 375, Lima 15081, Peru; laly.rojas@unmsm.edu.pe (L.A.R.R.); cgalvezr@unmsm.edu.pe (C.M.G.R.); mrequenam@unmsm.edu.pe (M.F.R.M.); rbocanegraa@unmsm.edu.pe (R.D.P.B.A.); rcallap@unmsm.edu.pe (R.D.C.P.); 2 Escuela de Formación Profesional de Odontología, Facultad de Odontología, Universidad Nacional Daniel Alcides Carrión (UNDAC), Av. Daniel Alcides Carrión s/n, Cerro de Pasco 19001, Peru; msalvatierrac@undac.edu.pe (M.A.S.C.); jbalcazarc@undac.edu.pe (J.N.B.C.)

**Keywords:** oral microbiome, dental caries, health-associated taxa, *Streptococcus anginosus*, *Rothia aeria*, Oxford nanopore sequencing, full-length 16S rRNA, ecological filter, dysbiotic convergence, Peruvian children

## Abstract

Dental caries is the most prevalent chronic noncommunicable disease in Peruvian schoolchildren (>70%). Full-length 16S Oxford Nanopore Technology (ONT) sequencing enables species-level resolution unachievable with conventional Illumina V3–V4 platforms, critical for resolving intrageneric diversity in the dominant oral genus Streptococcus This is a cross-sectional study (STROBE/STORMS) of 30 children aged 8–10 years (15 with caries, ceod/CPOD ≥ 1; 15 caries-free, ceod/CPOD = 0) from IE N°3036 José Andrés Rázuri, San Martín de Porres, Lima, with caries status assessed by clinical examination using ICDAS-II criteria and summarized using the ceod/CPOD indices. Full-length 16S sequencing (~1500 bp; 27F/1492R; SQK-16S024) was performed on MinION MK1B (MN40465), with Flongle AUB828 in two runs (Run 1: 21,651 PASS reads, Q = 12.50; Run 2: 3750 PASS reads, Q = 11.89). The pipeline used was wf-16s v1.2.0 (EPI2ME, NCBI 16S rRNA), while for statistics, Mann–Whitney U test, Fisher’s exact test (α = 0.05), and PERMANOVA (999 permutations) were used. In total, 126 species were identified in 63 genera and 5 phyla. Streptococcus salivarius was dominant (22.5%; 30/30). Twenty-six species were exclusive to the caries group, led by S. mutans (7/15, 46.7%; OR = ∞; raw *p* = 0.006) and *S. anginosus* (4/15, 26.7%; OR = ∞; raw *p* = 0.038), plus *Lancefieldella parvula*, *Veillonella infantium*, and *Actinomyces naeslundii*. Fifteen species were exclusive to the healthy group, including *Rothia aeria*, *Gemella* sp., *Aggregatibacter kilianii*, and *Bulleidia extructa*. None of these taxon-level differences survived Benjamini–Hochberg correction for the 126 species tested (all FDR-adjusted *p* > 0.45); these findings are therefore presented as exploratory candidates rather than confirmed differences. Alpha diversity (Shannon *p* = 0.507) and global beta diversity (PERMANOVA *p* = 0.215) did not differ; dysbiotic convergence was significant (intragroup Bray–Curtis: 0.322 vs. 0.414; *p* < 0.001). This is the first ONT full-length 16S characterization of the salivary microbiome in Peruvian children, simultaneously identifying cariogenic and health-associated species profiles. These findings suggest dietary nitrate supplementation and xylitol as candidate prebiotic strategies warranting future intervention studies to restore health-associated taxa in caries-free children.

## 1. Introduction

Dental caries is the most prevalent chronic noncommunicable disease globally, affecting >70% of schoolchildren in Peru [1]. The polymicrobial model—grounded in the ecological plaque hypothesis—has displaced the monoinfectious hypothesis centered on *Streptococcus mutans*: ecological dysbiosis, defined as the imbalance between cariogenic and health-associated taxa, drives lesion initiation and progression [2,3,4,5].

Conventional short-read platforms (Illumina, ~300 bp, V3–V4 regions) have critical limitations in resolving species within *Streptococcus*—the dominant oral genus in children—as their hypervariable regions are insufficient to discriminate intrageneric phylogenetic diversity [6]. Oxford Nanopore Technology (ONT) generates full-length 16S reads (>1400 bp, V1–V9), elevating taxonomic resolution to species level [7]. Emerging tools such as Savont [8], providing amplicon sequence variants (ASVs) for ONT, and Myloasm [9] (*Nature Biotechnology*, 2026) confirm that modern nanopore sequencing requires modern computational tools to maximize diagnostic potential.

Beyond the general case for long-read sequencing, species-level resolution is particularly consequential in the caries context because closely related *Streptococcus* species carry markedly different clinical implications: *S. mutans* and *S. sanguinis* are established cariogenic and health-associated taxa, respectively, while *S. anginosus*-group species are implicated in abscesses and non-carious pathology—distinctions that collapse into a single genus-level signal under short-read approaches. This unresolved gap in prior 16S surveys limits their utility for guiding ecological intervention design.

Saliva, rather than supragingival plaque, was selected as the sampling matrix for this study. Dental caries is fundamentally a biofilm-mediated disease, and plaque collected directly from lesion sites more closely reflects the structured microbial community involved in its initiation and progression; saliva instead captures a pooled, whole-mouth signal from multiple oral niches. We nonetheless prioritized saliva because it is a practical, non-invasive, and reproducible sampling method well suited to pediatric field studies conducted in a school setting, and because studies directly comparing the two matrices in children show that salivary taxa retain meaningful, if somewhat attenuated, associations with caries status relative to plaque [10]. We therefore regard the present salivary profiling as a first, hypothesis-generating step, complementary to rather than a substitute for future plaque-based confirmation.

A critical limitation of available oral microbiome studies is that they identify caries-associated taxa without simultaneously characterizing health-associated taxa—the candidate protective species eliminated by the cariogenic environment. Without knowing the health profile, ecological restoration interventions cannot be designed. This study addresses both profiles simultaneously in the first ONT full-length 16S characterization of the salivary microbiome in Peruvian children.

The objectives of this study were: (1) to characterize the salivary oral microbiome at the species level in 30 children (15 with caries/15 caries-free) using ONT full-length 16S sequencing; (2) to identify taxa exclusive to the caries group and taxa exclusive to the healthy group; (3) to analyze alpha and beta diversity and document dysbiotic convergence; and (4) to discuss evidence-based dietary and prebiotic strategies for ecological modulation of the cariogenic microbiome.

## 2. Materials and Methods

### 2.1. Study Design and Population

This was a cross-sectional analytical study following STROBE/STORMS guidelines. Thirty children aged 8–10 years from IE N°3036 José Andrés Rázuri, San Martín de Porres, Lima, Peru, were enrolled: 15 with caries (ceod/CPOD ≥ 1) and 15 caries-free (ceod/CPOD = 0). The cohort comprised 15 girls and 15 boys, balanced between groups (caries group: 7 girls/8 boys; caries-free group: 8 girls/7 boys) and evenly distributed across three age strata (8, 9, and 10 years; 10 children per age stratum, 5 per group). Caries status was assessed by clinical examination of all teeth and surfaces using ICDAS-II criteria; findings were summarized using the ceo-d index for primary dentition and the CPOD index for permanent dentition, and children were classified into the caries group when presenting ceo-d/CPOD ≥ 1 and into the caries-free group when presenting ceo-d/CPOD = 0. In the caries group, the ceo-d/CPOD component breakdown showed that all caries experience corresponded to untreated active carious lesions (C/c components; aggregate C = 35, c = 30 across the 15 children), with no missing (P) or filled (O) teeth recorded in either dentition; no participant presented restored teeth reflecting past, treated caries experience. Exclusion criteria included antibiotic therapy within 3 months prior to sampling, antiseptic mouthwash use, probiotic supplementation, fixed orthodontic appliances, and chronic systemic diseases (e.g., asthma) requiring ongoing medication. No a priori sample size calculation was performed; participants were recruited by convenience sampling. A post hoc sensitivity/minimum-detectable-effect analysis, based on a 20,000-iteration Monte Carlo simulation of a two-sided Fisher’s exact test (the test actually used for prevalence comparisons), indicated that with 15 participants per group and α = 0.05, a baseline prevalence of 10%, and OR = 3 (the effect size used for the a priori design calculation in Section 4.6), achieved power was only ~8.7%; an OR ≈ 15.7 would be required to reach 80% power at this baseline prevalence and sample size. Non-significant results, particularly for diversity metrics, should therefore not be interpreted as evidence of equivalence between groups. Ethical approval was granted by the Ethics Committee, Facultad de Odontología, UNMSM (N°039-CEI-FO-2024). Written informed consent was obtained from parents/guardians.

### 2.2. Sample Collection and DNA Extraction

Unstimulated whole saliva (~5 mL) was collected between 9:00 a.m. and 12:00 p.m., approximately 2–3 h after a standardized breakfast (6:00–7:00 a.m.) and school arrival (8:00 a.m.). Participants were instructed to abstain from food, beverages, and oral hygiene procedures during the 2 h preceding collection. Immediately before sampling, each participant rinsed the mouth with 10 mL of water for 60 s and expectorated completely; after a 5 min rest period, participants remained seated with the head slightly tilted forward, without speaking or chewing, and passively pooled saliva on the floor of the mouth, expectorating it approximately every 60 s into a sterile, DNase/RNase/human-DNA-free 50 mL polypropylene conical tube (Diamond Max Centrifuge Tubes, Globe Scientific, Paramus, NJ, USA) until ~5 mL was obtained. No EDTA or other preservative was added at collection. Tubes were capped, labeled, and immediately placed in a cooler with gel refrigerant packs for transport to the laboratory. Sample collection was performed by two dentists (C.G.R. and T.V.R.C.) and the study’s lead investigator (L.A.R.R.). Samples were processed within 2 h or stored at −20 °C. DNA was extracted using the Extracta Plus DNA kit (50 RXN, Cat. 95213-050, Quantabio, Beverly, MA, USA) with enzymatic pretreatment (lysis buffer containing Tris-HCl, 2 mM EDTA, and Triton X-100, lysozyme 20 mg/mL, 37 °C, 30 min). Purity was verified by spectrophotometry (NanoDrop ND-1000 (Thermo Fisher Scientific, Wilmington, DE, USA); A260/A280 ≥ 1.8).

### 2.3. 16S rRNA Amplification and ONT Sequencing

The 16S rRNA gene (~1500 bp) was amplified using universal primers 27F/1492R (AccuStart II ToughMix (Quantabio, Beverly, MA, USA); 35 cycles). Library preparation used the SQK-16S024 kit (Oxford Nanopore Technologies, Oxford, UK), with barcoding provided by its integrated 16S Barcode Primers (01–24). Base-calling and barcode demultiplexing were performed in real time using the integrated Dorado basecaller in MinKNOW v6.0.8 (basecaller version 7.4.12, Oxford Nanopore Technologies), using the high-accuracy model dna_r9.4.1_e8_hac@v3.3 (dna_r9.4.1_450bps_hac.cfg) with a minimum Q-score threshold of 9. Sequencing was performed on MinION MK1B (device ID: MN40465), Flongle flow cell AUB828, in two consecutive runs: Run 1 (12 August 2024): 21,651 PASS reads (79.2%; Q = 12.50; mean length 1296 bp; 70.0% ≥ 1400 bp; 95/126 active pores (75.4%); 5.02 h; 34.56 Mb); Run 2 (13 August 2024, same flow cell, second use): 3750 PASS reads (63.3%; Q = 11.89; mean length 1384 bp; 77.7% ≥ 1400 bp; 20/126 active pores (15.9%), −78.9%; 24.1 h; 9.02 Mb). Barcodes CU (bc08–bc12, Run 1) correspond to surface control samples from a separate study and were excluded from taxonomic analysis.

### 2.4. Bioinformatic Analysis

PASS reads from both runs were combined per barcode and processed using the wf-16s v1.2.0 pipeline (EPI2ME Labs, Oxford Nanopore Technologies) against the NCBI 16S rRNA reference database (database_set: ncbi_16s_18s), using default, unmodified workflow parameters: classifier = minimap2 (alignment-based, default option), read length filter min_len = 800 bp/max_len = 2000 bp, minimum percent identity = 95%, and minimum reference coverage = 90%; reads mapping to a reference but failing the identity or coverage thresholds were relabeled as unclassified, and an abundance threshold of 1 read was applied at the reporting stage. The wf-16s pipeline does not include a dedicated chimera-detection/removal step; no additional chimera filtering was performed. An abundance matrix of 126 species × 30 samples was constructed. Run 3 was excluded (4.5% PASS reads; mean Q 4.83). Rarefaction was performed at the minimum depth of 120 reads/sample prior to alpha diversity calculation. Raw sequencing data are publicly available at NCBI SRA (BioProject PRJNA1463303; BioSamples SAMN60145657–SAMN60145686; Submission SUB16189569).

### 2.5. Statistical Analysis

Alpha diversity indices (Shannon H′, observed richness, and Chao1) were calculated after rarefaction. Beta diversity was assessed using Bray–Curtis dissimilarity with PCoA and PERMANOVA (999 permutations; scipy, Python 3.11). Intergroup comparisons used, for each of the 126 species, the Mann–Whitney U test (bilateral; α = 0.05; trends: 0.05 < *p* ≤ 0.10) on relative abundance and Fisher’s exact test on presence/absence prevalence, yielding two independent families of 126 tests each (252 comparisons total). Taxon exclusivity was defined as presence in ≥1 sample of one group and absence in all samples of the other group (0/15). Given that 126 species were tested in each of the two test families described above (252 comparisons total), raw *p*-values were additionally corrected for multiple comparisons using the Benjamini–Hochberg false discovery rate (FDR) procedure, which was applied separately within each family of 126 tests (i.e., Mann–Whitney q-values were computed among the 126 Mann–Whitney tests, and Fisher q-values among the 126 Fisher tests); both raw and FDR-adjusted *p*-values (q) are reported. All taxon-level findings, including nominally significant raw *p*-values, are presented as exploratory, hypothesis-generating observations from this pilot dataset rather than confirmed statistically robust differences, consistent with the small per-group sample size (Section 4.6).

## 3. Results

### 3.1. Sequencing Performance

**Figure 1:**
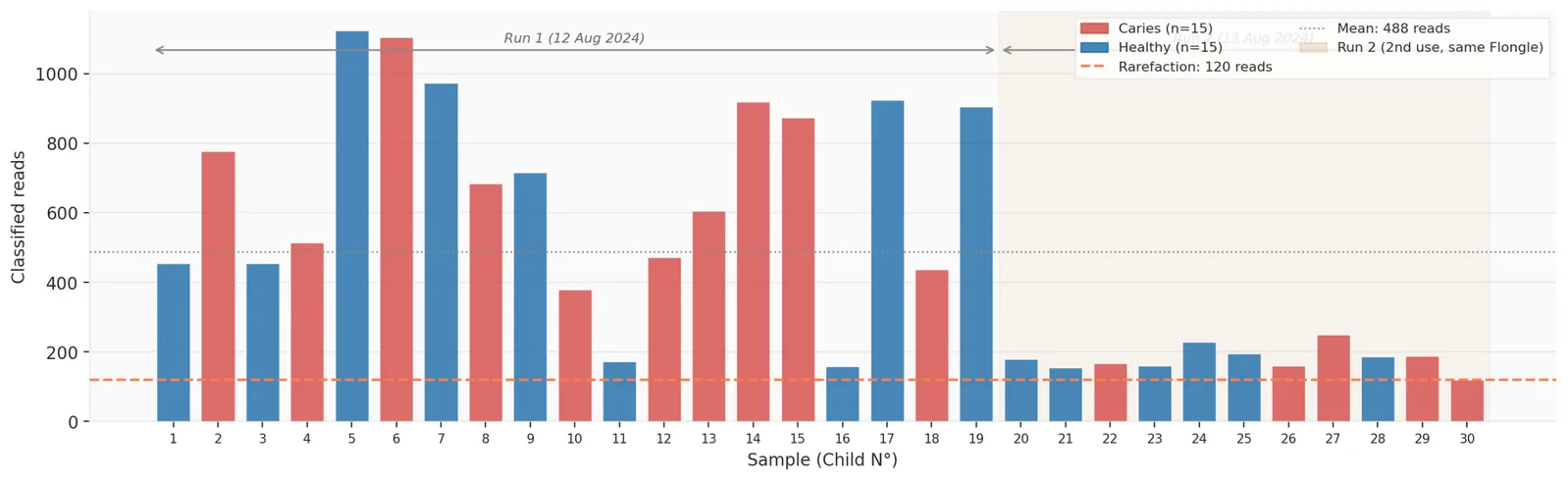
Sequencing depth per sample (classified reads). Orange dashed line = rarefaction threshold (120 reads min). Shaded area = Run 2 samples (second run, same Flongle flow cell).

Run 1 generated 21,651 PASS reads over 5.02 h (2.50; 95/126 active pores, 75.4%; 70.0% ≥ 1400 bp). Run 2 demonstrated marked flow cell degradation: 20/126 active pores (15.9%, −78.9% relative to Run 1) and 3750 PASS reads over 24.1 h (Q = 11.89; PASS rate 63.3%) (Figure 1). When combined, the total is 25,401 PASS reads across 30 samples (Table 1).

### 3.2. Global Taxonomic Composition

**Figure 2:**
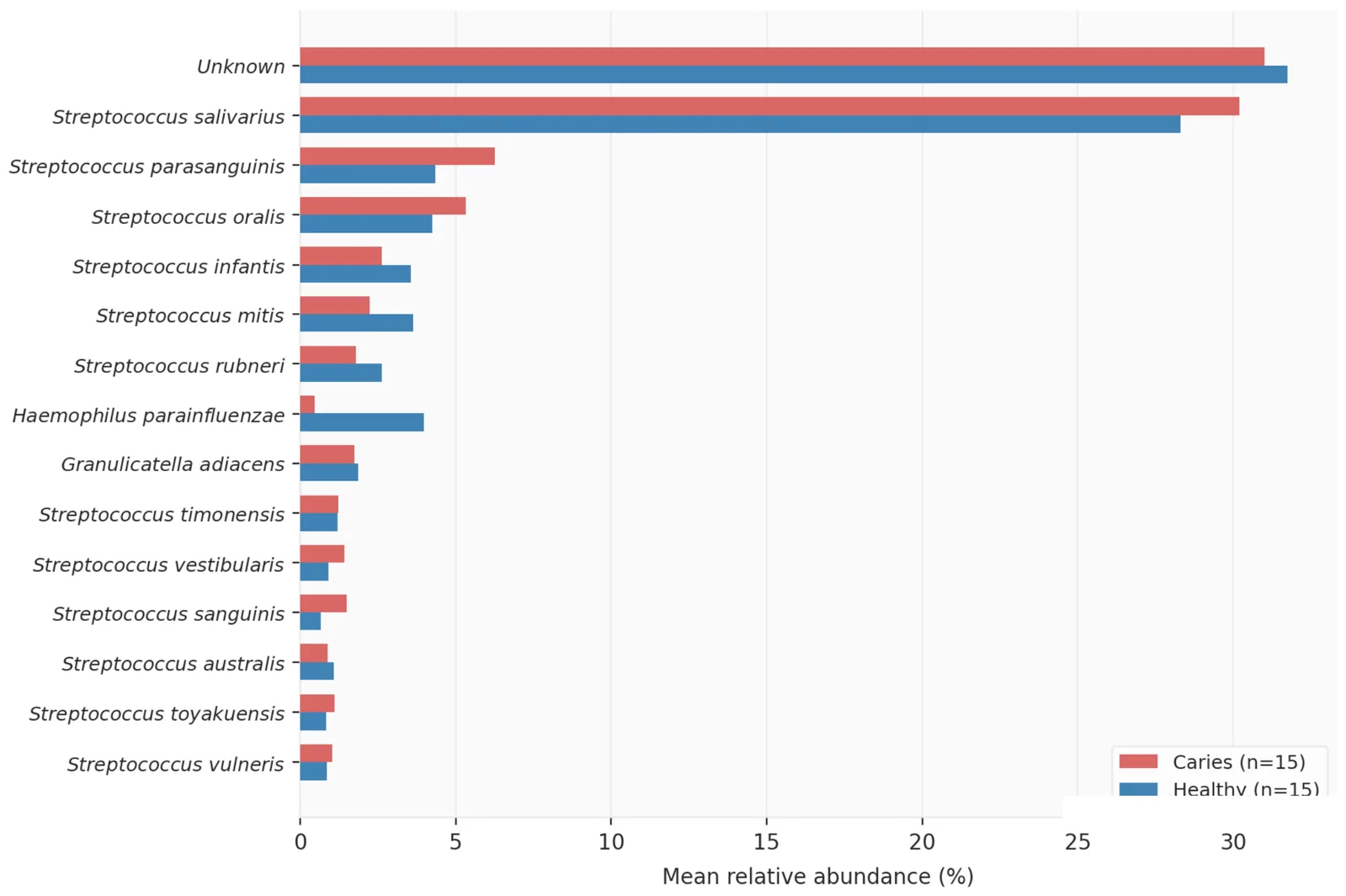
Top 15 species by mean relative abundance. Red = caries group; blue = healthy group. wf-16s v1.2.0, NCBI 16S rRNA; n = 30.

**Figure 3:**
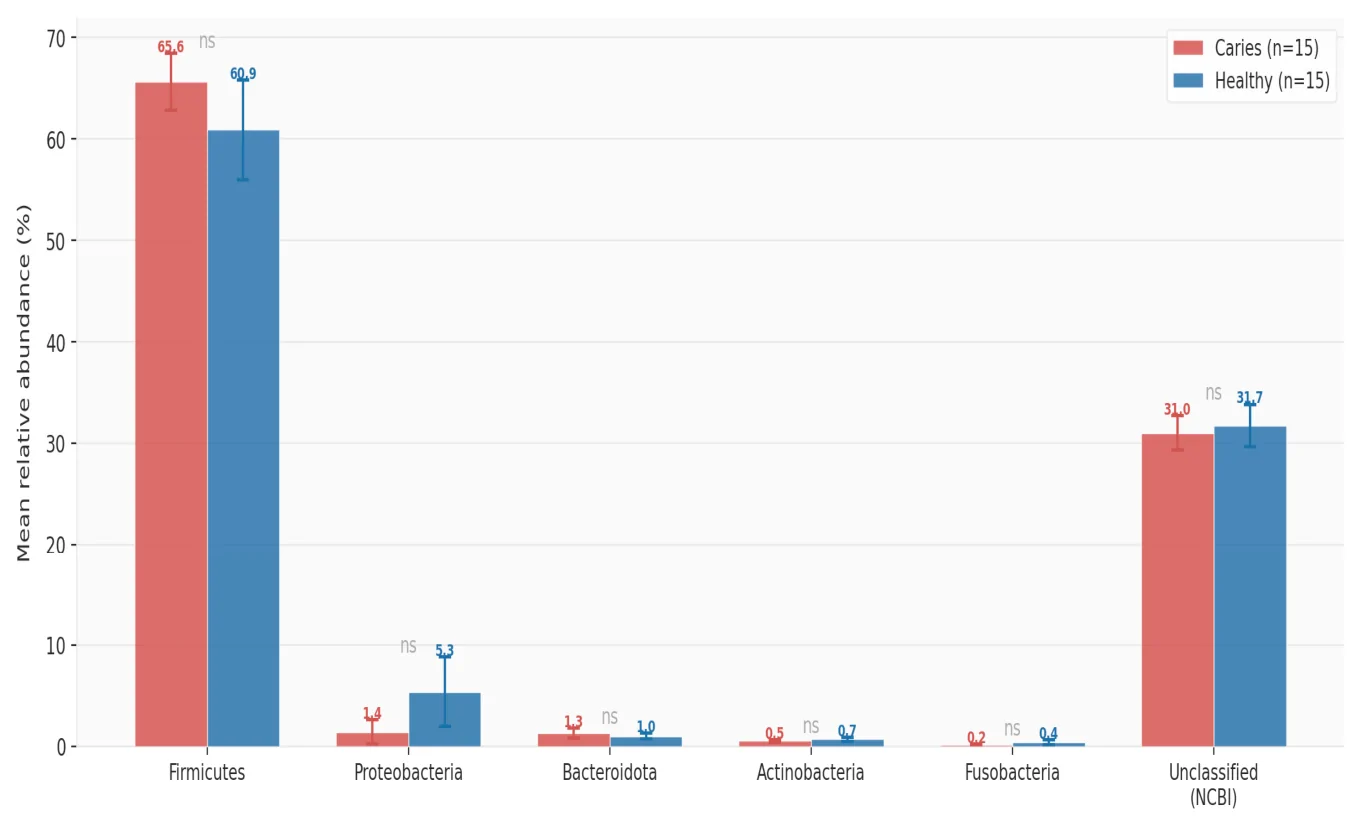
Phylum-level distribution by group (Mann–Whitney U; ns = not significant (*p* ≥ 0.05) for all phyla); 5 per group.

The combined analysis identified 126 species in 63 genera and 5 phyla (29.9% unclassified). Firmicutes was dominant in both groups without significant differences (Table 2). *Streptococcus salivarius* was the most abundant species (22.5%; 30/30), followed by *S. parasanguinis* (4.6%; 30/30) and *S. oralis* (3.9%; 30/30) (Figure 2 and Figure 3).

### 3.3. Alpha and Beta Diversity

**Figure 4:**
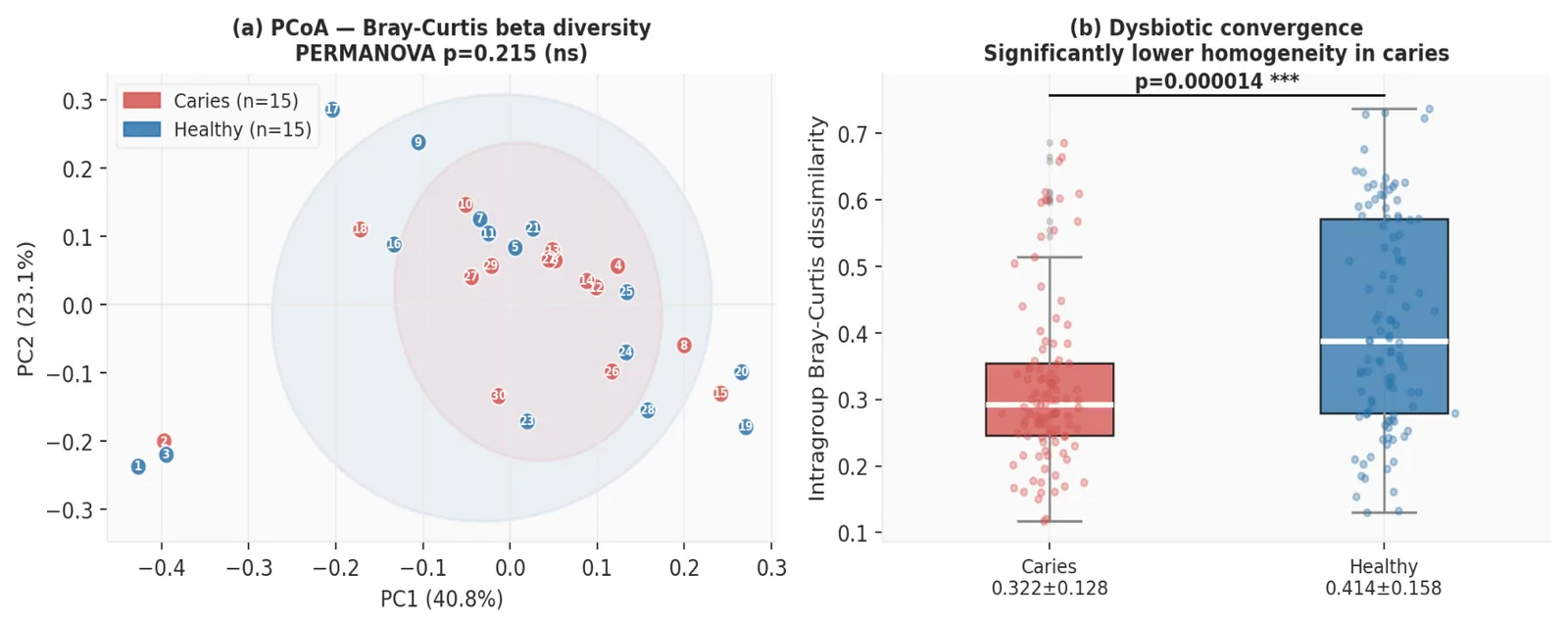
Beta diversity. (**a**) PCoA on Bray–Curtis dissimilarity (PERMANOVA *p* = 0.215, ns). (**b**) Dysbiotic convergence: intragroup Bray–Curtis dissimilarity significantly lower in caries (0.322 vs. 0.414; *** *p* < 0.001).

No significant differences in alpha diversity were observed: Shannon H′ = 2.109 ± 0.392 (caries) vs. 1.985 ± 0.450 (healthy; *p* = 0.507); observed richness 32.8 ± 14.1 vs. 30.1 ± 10.1 (*p* = 0.835). PERMANOVA on Bray–Curtis distances showed no global compositional separation (*p* = 0.215; R^2^ = 0.034) (Table 3, Figure 4). However, intragroup dissimilarity was significantly lower in the caries group (0.322 ± 0.128 vs. 0.414 ± 0.158; *p* < 0.001), evidencing statistically significant dysbiotic convergence.

### 3.4. Taxa with Nominal Differences Between Groups (Unadjusted for Multiple Comparisons)

**Figure 5:**
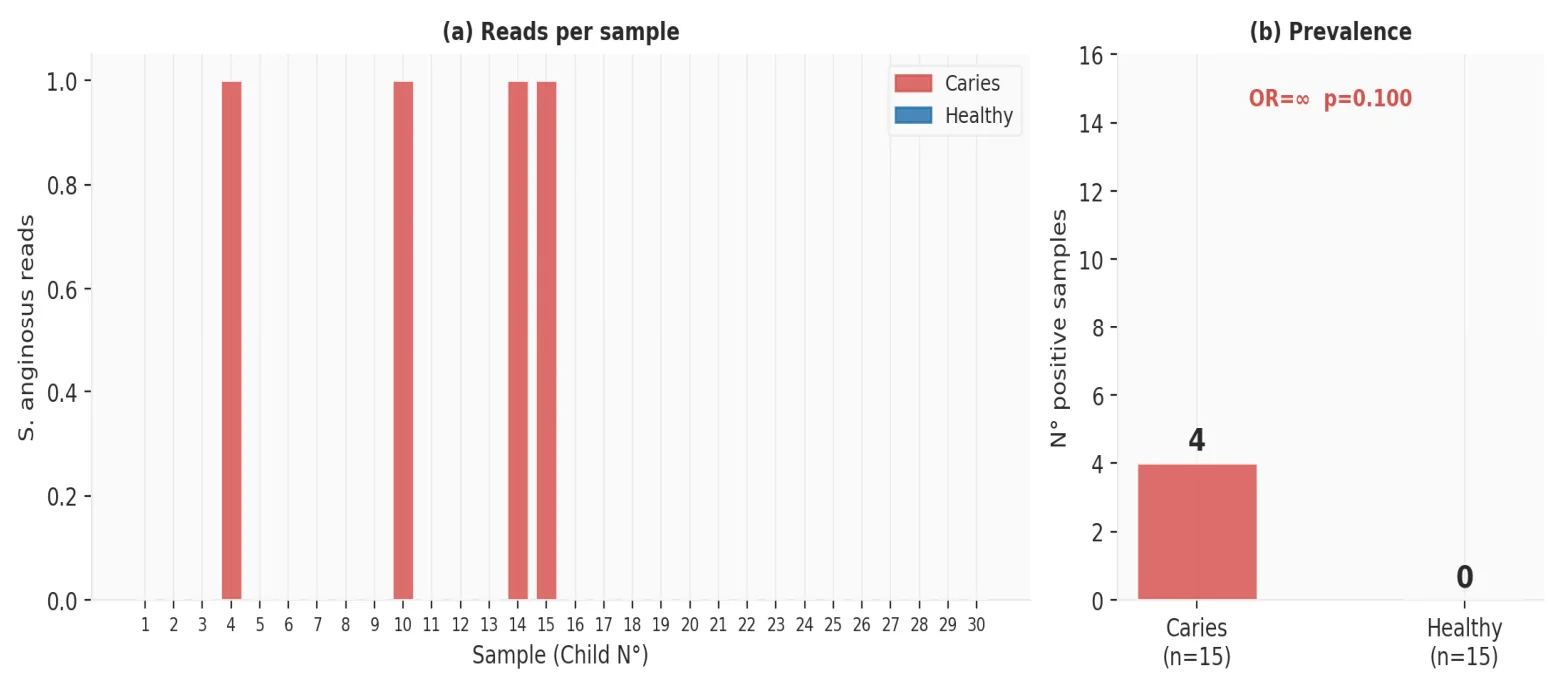
*S. anginosus*—exclusive to the caries group. (**a**) Reads per sample. (**b**) Prevalence comparison (4/15 vs. 0/15; OR = ∞; *p* = 0.038, ns = not significant (*p* ≥ 0.05). Mann–Whitney).

*S. mutans* showed the lowest raw *p*-value of all taxa tested: 7/15 children with caries (46.7%) vs. 0/15 healthy children (OR = ∞; raw *p* = 0.004 Mann–Whitney, *p* = 0.006 Fisher; FDR-adjusted *p* = 0.454 and 0.794, respectively—not significant after correction). *Veillonella dispar* was more abundant in caries at the raw significance threshold (11/15 vs. 4/15; OR = 7.56; raw *p* = 0.017; FDR-adjusted *p* = 0.701, not significant). *S. anginosus* was detected exclusively in the caries group (4/15, 26.7% vs. 0/15; OR = ∞; raw *p* = 0.038 Mann–Whitney; FDR-adjusted *p* = 0.775, not significant), a species-level distinction that is only possible with ONT full-length resolution and constitutes the most novel exploratory candidate identified here (Table 4, Figure 5), pending confirmation in a larger cohort.

### 3.5. Taxa Exclusive by Group: Cariogenic and Oral Health Profiles

**Figure 6:**
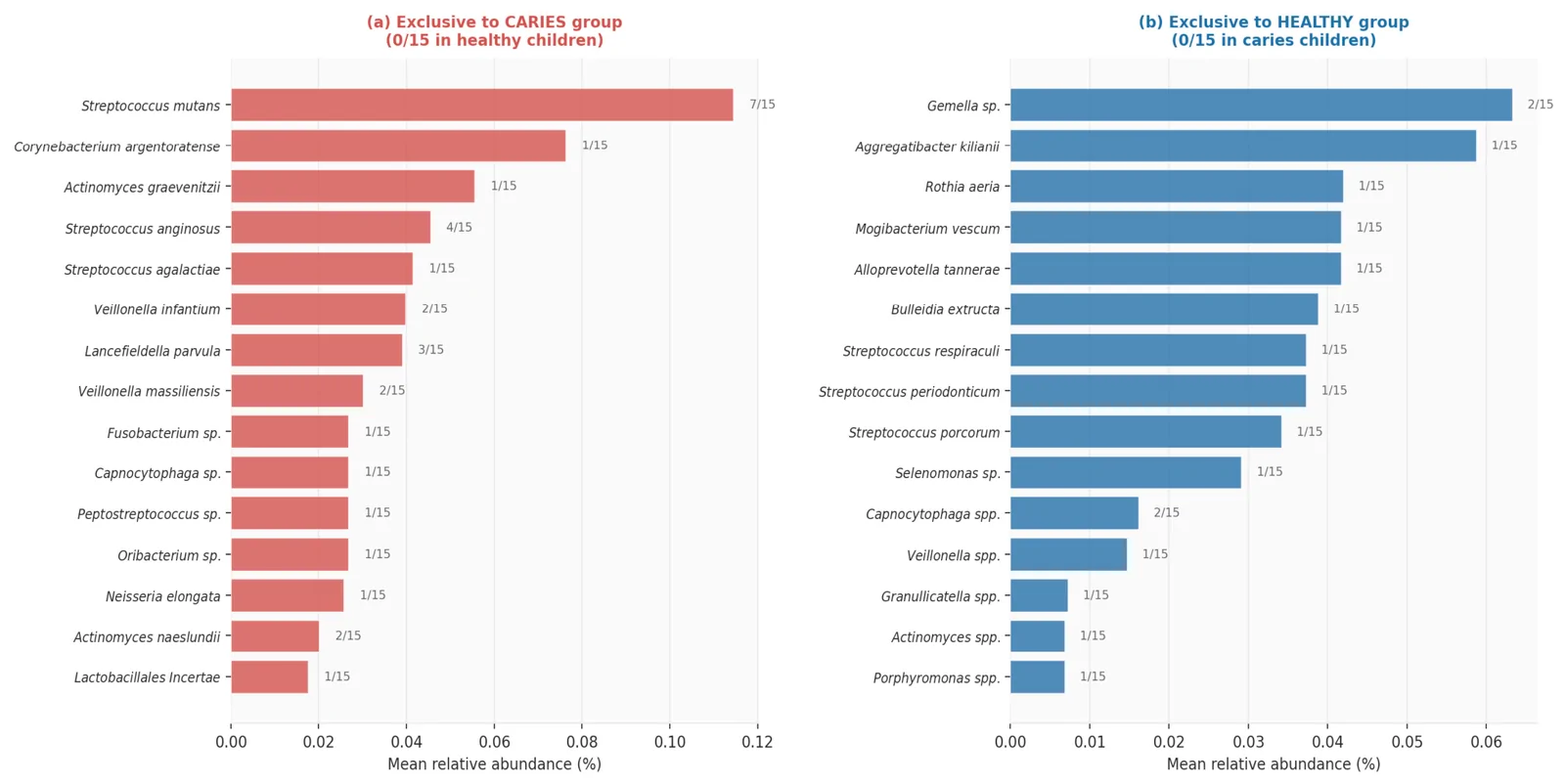
Taxa exclusive by group: (**a**) 26 species exclusive to caries group (red); (**b**) 15 species exclusive to healthy group (blue). Values to the right = prevalence.

**Figure 7:**
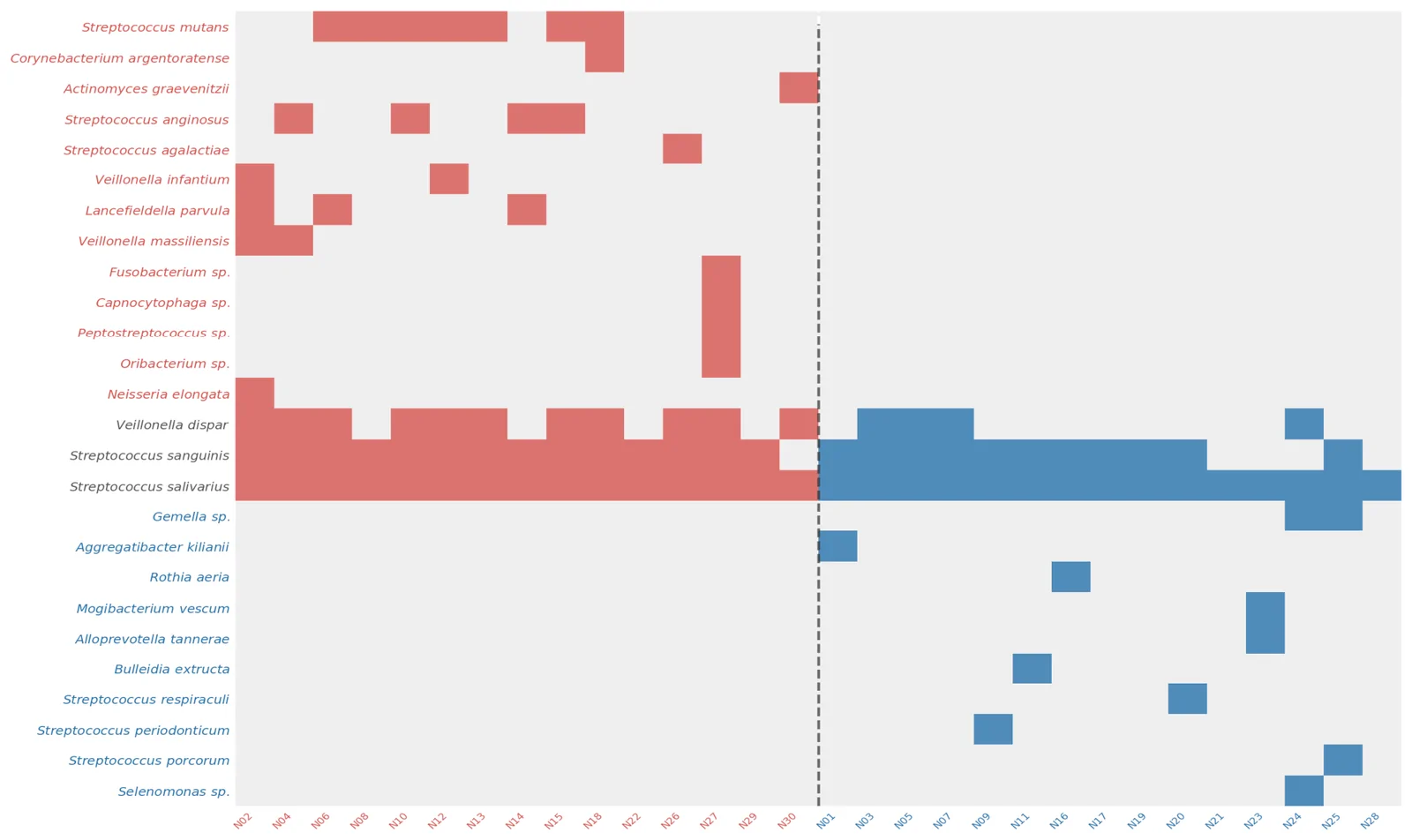
Presence/absence heatmap. Red/bold = exclusive to caries (n = 15); blue/bold = exclusive to healthy group (n = 15); gray = absent. ONT full-length 16S; n = 30.

The exclusivity analysis revealed 26 species exclusive to the caries group and 15 species exclusive to the healthy group, constituting the first cariogenic dysbiotic profile and oral health profile documented by ONT full-length 16S in Peruvian children (Figure 6 and Figure 7, Table 5 and Table 6). We use the term “exclusive” strictly in its operational, descriptive sense defined in Section 2.5 (detected in ≥1 sample of one group and absent in all 15 samples of the other); with only 15 individuals per group, most of these taxa were detected in a single participant (1/15), so these patterns should be read as candidate, exploratory associations that may partly reflect individual variability rather than confirmed group-specific biological signatures, pending validation in larger cohorts.

## 4. Discussion

### 4.1. Global Composition and Core Microbiome

The dominance of Firmicutes and *Streptococcus* is consistent with the international literature [11]. *S. salivarius* as the dominant species (22.5%; 30/30) reflects the expected early colonizer profile, inhibiting acidogenic species through bacteriocin production. The absence of phylum-level differences between groups confirms that caries modifies species-level composition without altering global taxonomic structure.

### 4.2. The Cariogenic Ecological Filter

The significant dysbiotic convergence (intragroup Bray–Curtis: 0.322 in caries vs. 0.414 in healthy; *p* < 0.001)—without global alpha diversity loss—quantifies the cariogenic ecological filter. The mechanism is dual: (1) pH reduction below 5.5 alters bacterial community structure, with ~60% of oral taxa contributing to the shift from neutral to acidic microenvironments [12]; (2) *S. mutans* produces mutacins inhibiting diverse Gram-positive bacteria, creating a positive feedback cycle consolidating cariogenic dysbiosis [13].

### 4.3. Cariogenic Dysbiotic Profile

*S. mutans* (46.7%; raw *p* = 0.006, ns after FDR correction) is consistent with its central acidogenic role documented elsewhere. *S. anginosus* (26.7%; raw *p* = 0.038, ns after FDR correction), a taxon of the anginosus group (SAG), has been associated with oral abscesses and pathological biofilms; species-level identification—impossible with V3–V4 short-read platforms—was enabled by ONT full-length resolution and represents the most novel exploratory candidate identified here, pending confirmation in a larger cohort. *Lancefieldella parvula* and *Veillonella infantium* reinforce the polymicrobial model, converting lactate into short-chain acids [12]. *Actinomyces naeslundii* is a recognized early colonizer in root caries [3].

### 4.4. Oral Health Profile and Protective Mechanisms

*Rothia aeria* is a member of the health-associated Rothia genus, whose species have recently become genetically tractable via transposon mutagenesis, opening avenues for future mechanistic studies [14]; a related species, *R. mucilaginosa*, produces enterobactin, a metal-chelating siderophore that reduces *S. mutans* growth in co-culture studies [15]; whether R. aeria specifically antagonizes S. mutans via a comparable mechanism has not, to our knowledge, been established in the peer-reviewed literature and remains an open question for future co-culture studies. *Gemella* sp. is among the ten most frequently detected taxa in healthy oral microbiomes [11]. *Aggregatibacter kilianii* has been reported in healthy periodontal microbiota, and *Bulleidia extructa* is a recognized commensal of healthy oral mucosa [16].

These findings should be interpreted in the context of the sampling site. Saliva reflects a pooled, whole-mouth signal rather than the tooth-adjacent biofilm where caries lesions initiate; studies directly comparing saliva with supragingival plaque in children report lower microbial diversity in saliva and a distinct community structure between the two niches, with cariogenic taxa such as *S. mutans* and *V. dispar* more strongly discriminating caries status in plaque than in saliva [10]. Saliva nonetheless remains a practical, non-invasive matrix for pediatric cohorts and captures the taxa exclusive to each group identified here, though supragingival plaque may offer greater discriminatory power in future validation studies. Regarding age, pediatric oral microbiomes differ modestly from adult microbiomes, with children showing higher relative abundances of early-colonizing, health-associated taxa and lower abundances of periodontal pathogens such as *Tannerella forsythia* and *Porphyromonas gingivalis*, consistent with a microbiome still dominated by early colonizers [17]. This developmental context supports interpreting the cariogenic and health-associated profiles identified here as specific to the pediatric oral ecosystem rather than a direct downscaling of adult caries microbiology.

Beyond the taxa formally exclusive to each group, several additional species showed a numerically higher relative abundance in the healthy group without reaching statistical significance in this cohort: *Haemophilus parainfluenzae* (3.97% vs. 0.46%; *p* = 0.645), *Streptococcus mitis* (3.64% vs. 2.23%; *p* = 0.229), *Streptococcus infantis* (3.56% vs. 2.63%; *p* = 0.803), and *Streptococcus rubneri* (2.63% vs. 1.80%; *p* = 0.442; all Mann–Whitney, ns). While these trends cannot be interpreted as confirmed differences given the sample size, they are compatible with mechanistic evidence of protective roles reported elsewhere: *H. parainfluenzae* is a nitrate-reducing bacterium that generates NH_3_, raises local pH, and antagonizes aciduric species [18]; *S. mitis* has been shown to inhibit *S. mutans* biofilm formation through pyruvate-oxidase-dependent hydrogen peroxide production [19]. At the phylum level, Proteobacteria—which includes the nitrate-reducing genera *Neisseria* and *Haemophilus*—was likewise nominally higher in the healthy group (5.73% vs. 1.31%; *p* = 0.656, ns; Table 2). Although this difference did not reach significance, it is compatible with the hypothesis that habitual dietary nitrate intake favors nitrate-reducing Proteobacteria and thereby an alkalinizing, less cariogenic oral environment (Section 4.5); larger cohorts are needed to test this hypothesis formally.

### 4.5. Strategies to Preserve Health-Associated Taxa

Dietary nitrate—concentrated by salivary glands to 5–8 mM after vegetable-rich meals—increased *Neisseria* (3.1×) and *Rothia* (2.9×) within 5 h of exposure while reducing cariogenic *Veillonella* and *Streptococcus*; additionally, it elevated salivary pH, directly limiting caries development [20,21]. Dietary sources for Peruvian schoolchildren include beetroot, spinach, lettuce, and chard. Xylitol acts through the futile phosphate cycle, reducing *S. mutans* selectively, with demonstrated preventive efficacy in children [22]. A combined strategy—reduction of fermentable carbohydrates + dietary nitrate + school xylitol—represents an ecologically informed intervention implementable within Peru’s MINSA/MINEDU school health programs.

### 4.6. Technical Considerations and Limitations

The main limitation of this pilot study is its modest sample size (n = 30; 15 per group), which limits statistical power for low-prevalence taxa and constrains the generalizability of the findings. Oral hygiene habits were not systematically recorded, precluding assessment of their potential effect on salivary microbiome composition; future studies should incorporate these variables and standardize the interval between the last toothbrushing and sample collection. The protocol excluded participants with systemic diseases and considered recent antibiotic use; however, information on other medications with potential hyposalivatory effects was not systematically collected and should be addressed in future studies. Dedicated extraction blanks, PCR negative controls, mock community standards, and positive sequencing controls were not included in this pilot dataset; low-prevalence taxa (1–2/15), including several taxa reported as group-exclusive, should accordingly be interpreted as preliminary observations rather than confirmed group-specific biological markers, pending validation with appropriate controls in future studies. The 29.9% of reads left unclassified against the generic NCBI database represents a further bioinformatic limitation—reducible to <5% with HOMD v4.2 (reprocessing underway). The second Flongle flow cell use documented 78.9% pore loss (95 → 20 active pores), providing practical benchmarking for resource-limited settings. Low individual prevalence of health-associated taxa (1–2/15) reflects the pilot study; based on the same Monte Carlo Fisher’s exact framework described in Section 2.1, validation of an OR = 3 effect at a baseline prevalence of 30% would require approximately n ≈ 61 per group (~122 total) for 80% power (α = 0.05, two-sided). Savont [8] (ASVs for ONT) represents the next methodological step to maximize taxonomic resolution.

## 5. Conclusions

This is the first ONT full-length 16S characterization of the salivary microbiome in Peruvian children (n = 30), simultaneously identifying a cariogenic dysbiotic profile (26 exclusive species) and an oral health profile (15 exclusive species) undetectable with short-read platforms. *S. anginosus* (raw *p* = 0.038) and *S. mutans* (raw *p* = 0.004) emerged as caries-exclusive candidate taxa in this exploratory analysis, though neither difference remained significant after correction for the 126 comparisons performed. *Rothia aeria*, *Gemella* sp., and *Aggregatibacter kilianii* emerge as candidate health-associated taxa whose absence in the caries group is associated with the cariogenic acid environment, a mechanistic interpretation that warrants direct testing rather than a demonstrated causal effect. Dysbiotic convergence without alpha diversity loss (Bray–Curtis: 0.322 vs. 0.414; *p* < 0.001) confirms the focal polymicrobial dysbiosis model, documented for the first time in a Latin American pediatric cohort. These candidate health-associated taxa and the dysbiotic convergence pattern identified here support dietary nitrate and xylitol as biologically plausible, hypothesis-generating prebiotic strategies whose efficacy in restoring protective taxa should be tested in future intervention trials in caries-free children.

## Figures and Tables

**Table 1:** Sequencing performance metrics by run.

Parameter	Run 1	Run 2
PASS reads (%)	21,651 (79.2%)	3750 (63.3%)
Mean Q score	12.50	11.89
Mean length (PASS)	1296 bp	1384 bp
Reads ≥ 1400 bp	15,150 (70.0%)	2915 (77.7%)
Active pores/126	95/126 (75.4%)	20/126 (15.9%)
Duration	5.02 h	24.1 h
Total bases (PASS)	34.56 Mb	9.02 Mb

**Table 2:** Mean relative abundance (%) by phylum according to oral health status.

Phylum	Caries (n = 15)	Healthy (n = 15)	*p* Value
Firmicutes	64.59 ± 11.4%	61.75 ± 19.4%	0.712 (ns)
Proteobacteria	1.31 ± 4.5%	5.73 ± 13.7%	0.656 (ns)
Bacteroidota	0.89 ± 0.9%	1.12 ± 1.4%	0.548 (ns)
Actinobacteria	0.51 ± 0.5%	0.61 ± 0.6%	0.623 (ns)
Fusobacteria	0.18 ± 0.3%	0.29 ± 0.5%	0.541 (ns)
Unclassified (NCBI)	32.5 ± 7.2%	30.5 ± 6.9%	0.487 (ns)

Mann–Whitney U, two-sided. ns = not significant.

**Table 3:** Alpha and beta diversity indices by group (rarefaction: 120 reads/sample).

Index	Caries (n = 15)	Healthy (n = 15)	*p* Value
Shannon (H′)	2.109 ± 0.392	1.985 ± 0.450	0.507 (ns)
Observed richness	32.8 ± 14.1	30.1 ± 10.1	0.835 (ns)
Chao1	59.2 ± 42.7	62.3 ± 38.8	0.787 (ns)
BC intragroup (mean ± SD)	0.322 ± 0.128	0.414 ± 0.158	*p* < 0.001 ***
PERMANOVA (global BC)	—	—	0.215 (ns)

*** *p* < 0.001. ns = not significant (*p* ≥ 0.05), — = not applicable. Mann–Whitney U, two-sided. BC = Bray–Curtis.

**Table 4:** Taxa with nominal between-group differences before multiple-testing correction (raw *p* < 0.10).

Taxon	Caries %	Healthy %	*p* (MW)	*p* (Fisher)	Prev C	Prev H/OR
*S. mutans*	0.114	0.000	0.004 **	0.006 **	7/15 (46.7%)	0/15/∞
*V. dispar*	0.577	0.129	0.017 *	0.027 *	11/15 (73.3%)	4/15/7.56
*S. sanguinis*	1.495	0.672	0.014 *	ns	14/15 (93.3%)	11/15/5.09
*S. anginosus*	0.045	0.000	0.038 *	0.100 †	4/15 (26.7%)	0/15/∞
*P. pasteri*	0.064	0.168	0.152	0.245	3/15 (20.0%)	7/15/0.29

** *p* < 0.01; * *p* < 0.05; † *p* < 0.10; ns = not significant. Benjamini–Hochberg q-values (each test family corrected separately across all 126 species): *S. mutans* q (MW) = 0.454, q (Fisher) = 0.794; *V. dispar* q (MW) = 0.701, q (Fisher) = 1.000; *S. sanguinis* q (MW) = 0.701, q (Fisher) = 1.000; *S. anginosus* q (MW) = 0.775, q (Fisher) = 1.000; *P. pasteri* q (MW) = 0.775, q (Fisher) = 1.000. None remain significant after correction; raw *p*-values above are presented for transparency but should be interpreted as exploratory. Raw and FDR-adjusted *p*-values (q) for all 126 species are provided in Supplementary Table S1.

**Table 5:** Species exclusive to the caries group (n = 26; absent in all 15 healthy children).

Species	Prev (n/15)	Mean %	*p* Fisher	Phylum
*S. mutans*	7/15	0.1144	0.006 **	Firmicutes
*S. anginosus*	4/15	0.0454	0.100 †	Firmicutes
*Lancefieldella parvula*	3/15	0.0390	0.224	Actinobacteria
*Veillonella infantium*	2/15	0.0398	0.483	Firmicutes
*Veillonella massiliensis*	2/15	0.0301	0.483	Firmicutes
*Actinomyces naeslundii*	2/15	0.0201	0.483	Actinobacteria
*Enterococcus* spp.	2/15	0.0171	0.483	Firmicutes
*Corynebacterium argentoratense*	1/15	0.0763	1.000	Actinobacteria
*Porphyromonas endodontalis*	1/15	0.0153	1.000	Bacteroidota
Other 17 species	1/15 each	<0.010	ns	Various

** *p* < 0.01; † *p* < 0.10; ns = not significant. Fisher’s exact test, two-sided. After Benjamini–Hochberg FDR correction across all 126 species tested, no taxon in this table remains statistically significant (minimum FDR-adjusted *p* = 0.794, for *S. mutans*); these taxa should be interpreted as exploratory candidates for group exclusivity given the small per-group sample size, not confirmed biological markers. Full raw and FDR-adjusted (q) values for all 126 species, for both test families, are provided in Supplementary Table S1.

**Table 6:** Species exclusive to the healthy group (n = 15; absent in all 15 children with caries).

Species	Prev (n/15)	Mean %	*p* Fisher	Phylum
*Gemella* sp.	2/15	0.0633	0.483	Firmicutes
*Aggregatibacter kilianii*	1/15	0.0587	1.000	Proteobacteria
*Rothia aeria*	1/15	0.0419	1.000	Actinobacteria
*Alloprevotella tannerae*	1/15	0.0417	1.000	Bacteroidota
*Bulleidia extructa*	1/15	0.0388	1.000	Firmicutes
*S. respiraculi*	1/15	0.0372	1.000	Firmicutes
*Capnocytophaga* spp.	2/15	0.0162	0.483	Bacteroidota
Other 8 species	1/15 each	<0.010	ns	Various

Low individual prevalence (1–2/15) reflects the pilot study design; validation of comparable effect sizes would require substantially larger cohorts (see Section 4.6 for the sensitivity/minimum-detectable-effect analysis). As with Table 5, none of these taxa remain significant after Benjamini–Hochberg FDR correction and should be interpreted as exploratory candidates (see Supplementary Table S1 for full raw and q-values).

## Data Availability

Raw sequencing data are publicly available at NCBI SRA: BioProject PRJNA1463303; BioSamples SAMN60145657–SAMN60145686; Submission SUB16189569.

## Supplementary Materials

The following supporting information can be downloaded at: https://www.mdpi.com/article/10.3390/microorganisms14091897/s1, Table S1: Raw and Benjamini-Hochberg FDR-adjusted *p*-values (Mann-Whitney and Fisher’s exact tests) for all 126 species identified, comparing caries and caries-free groups.
